# A Case of Brainstem Anesthesia after Retrobulbar Block for Globe Rupture Repair

**DOI:** 10.1155/2021/2619327

**Published:** 2021-12-13

**Authors:** Tavish Nanda, Lisa Ross, Gregory Kerr

**Affiliations:** ^1^Department of Ophthalmology, NYC Health+Hospitals/Harlem, New York, NY, USA; ^2^Department of Ophthalmology, Columbia University Irving Medical Center, New York, NY, USA; ^3^Department of Anesthesia, NYC Health + Hospitals/Harlem, New York, NY, USA

## Abstract

**Purpose:**

To present a rare case of brainstem anesthesia from retrobulbar block and discuss evidence-based methods for reducing the incidence of this complication.

**Case:**

A 72-year-old female, was given a retrobulbar block of 5 mL of bupivacaine 0.5% for postoperative pain management, after a globe rupture repair under general anesthesia. Prior to injection, the patient was breathing spontaneously via the anesthesia machine circuit and had not received any additional narcotics/muscle relaxants for 2.5 hr (with full recovery of neuromuscular blocking agent after anesthetic reversal). Over 7 min, however, there was a steady increase in ETCO_2_ and the patient became apneic, consistent with brainstem anesthesia. She remained intubated and was transported to the postanesthesia care unit for prolonged monitoring, with eventual extubation. *Discussion*. Brainstem anesthesia is an important complication to recognize as it can lead to apnea and death. The judicious use of anesthetic volume, shorter needle tips, and mixed formulations can help reduce the chance of brainstem anesthesia. Observation of the contralateral eye 5–10 minutes after injection for pupillary dilation, and prior to surgical draping, can help identify early CNS involvement.

## 1. Introduction

A retrobulbar (RB) block usually involves a mix of lidocaine 2% and bupivacaine 0.5–0.75% injected into the intraconal space. Other variations include 4% lidocaine, 2% mepivacaine, epinephrine 1 : 1000, and 5–150 IU hyaluronidase. An RB block impacts the ciliary ganglion (pupil dilation and corneal/conjunctival anesthesia) and causes paralysis of the 3^rd^ and 6^th^ cranial nerves. The use of this technique has dropped significantly in favor of topical, peribulbar, and blunt subtenons injections. In modern practice, RB blocks account for only 0.5% of regional anesthesia used in cataract surgery and are utilized more for early surgical trainees [1]. In other surgical conditions, however, such as conjunctival melanoma, retrobulbar anesthesia is used almost exclusively to prevent ballooning of the conjunctiva, which can hamper complete tumor excision [2]. Due to the nature of this injection, rare vision-threatening and life-threatening complications can occur, including retrobulbar hemorrhage, cardiac arrhythmia, and brainstem anesthesia (BSA) [1, 3]. In this report, we present a case of brainstem anesthesia after retrobulbar block and review methods for early identification and prevention of this life-threatening complication.

## 2. Case Description

A 72-year-old female presented to the emergency room after injuring her left eye during a syncopal episode. A computed tomography scan performed in the emergency department revealed disorganization of the left globe with blood in the vitreous humor. Her medical history included hypertension, systemic lupus erythematosus, and coronary artery disease.

Her vital signs were within normal limits with the exception of mild systolic hypertension. She underwent immediate globe rupture repair under general anesthesia with successful reapproximation of a large 20 mm scleral laceration. In the immediate postoperative period, rather than performing subtenon injection due to the possibility of a more posterior rupture, the surgeon chose to perform a retrobulbar block using 5 mL of bupivacaine 0.5% for postoperative pain management and akinesis (to prevent extrusion of additional intraocular contents). This was done using a 31 mm 25-gauge blunt needle.

No blood was aspirated back into the syringe, and the needle advanced without incident. Prior to injection, the patient was breathing spontaneously via the anesthesia machine circuit and had not received any additional narcotics/muscle relaxants for 2.5 hours. There was full recovery of neuromuscular blocking agent after reversal, as demonstrated by the use of a nerve stimulator. Over a duration of 7 minutes, however, the anesthesiologist noted a steady increase in end-tidal CO_2_, resulting in apnea. She remained intubated and was transported to the postanesthesia care unit for an additional 1.5 hours, after which she was successfully extubated. She was admitted to medicine and monitored for an additional time of 48 hours. No new neurologic or cardiac deficits were found, and she was discharged without event.

## 3. Discussion

There are two theorized mechanisms for BSA from retrobulbar block. The first, and most commonly accepted mechanism, is accidental puncture of the optic nerve sheath, with retrograde flow to the brainstem regions [3]. Intrasheath injection of the optic nerve can occur with upward deviation of the eyeball, which bends the optic nerve inferiorly and into contact with the advancing needle [4]. Evidence for this mechanism is supported by cadaveric studies, in which radio-opaque dye injected into the intraorbital subdural space was found in the midbrain [5, 6]. In vivo evidence was found in another case of suspected BSA, in which lidocaine and bupivacaine were recovered in the cerebrospinal fluid on subsequent spinal tap [7]. Usually, intravenous lidocaine levels of 5 *µ*g/ml (2.1–4.5 *µ*g/ml of bupivacaine) are required to cause CNS depression and apnea. In none of the published cases in which serum levels were reported did patients reach these thresholds [8, 9]. It is thought that levels in the immediate region of localized spread are high enough to induce serious complications, while serum levels may reflect a more diluted concentration [10].

The second mechanism involves inadvertent injection of the ophthalmic artery or smaller intraorbital tributaries with forced, retrograde flow. In up to 15% of patients, an anomalous inferior ophthalmic artery can course near the optic nerve, increasing the risk for arterial injection [11]. The differentiating factor between intra-arterial and intrasheath injection appears to be (1) the speed of symptoms and (2) the development of seizure activity in the former [12, 13]. Intra-arterial injection usually causes symptoms seconds after injection, while BSA from a nerve sheath injection occurs over a matter of minutes (5–50 min, avg. 20.5 min, in the published literature) [3, 8, 14–18]. Intra-arterial injection of lidocaine, for example, reaches the carotid arteries in just 15 seconds [10].

Although BSA is extremely rare, occurring in less than 0.27–1.5% of patients (Table 1), misidentification can result in respiratory arrest and death.

Early signs of brainstem involvement can be subtle. Symptoms, such as aphasia, confusion, dysphagia, shivering, hypotension, bradycardia, and hypertension, may be overlooked in a postsurgical ambulatory setting, where patients do not have immediate access to emergency care or frequent monitoring [15]. When identified and treated, however, the patient rarely has any residual side effects after full recovery, usually within 24–48 hours [1, 3, 18].

There are several methods for minimizing the risk of BSA. In the preoperative phase, placing the block prior to surgical draping can allow for assessment of the contralateral eye before obscuring the patient's face from view. Retrograde flow would affect the chiasm first, resulting in contralateral paresis or pupillary dilation. Although chiasmal involvement is not predictive of respiratory depression, its presence can alert the surgical team to the possibility of an impending complication [14, 20].

Another method for risk reduction involves having the patient maintain a central, steady gaze during injection. The historical Atkinson's method, which advised an “up and in” gaze in order to move the inferior oblique and inferior rectus complex out of the path of the needle, was originally practiced before several studies demonstrated undue risk to the optic nerve [21, 22]. Ahn et al., for example, noted that the use of the historical Atkinson's position appeared to be associated with a higher rate of BSA in their series, prior to an adjustment in technique [16].

Other authors have endorsed a “down and out” position, which directs the needle parallel to a taut and straightened optic nerve, though this allows the patient to visualize the incoming needle [19, 23]. A central position may be the most reasonable option, as this avoids kinking of the nerve and vasculature towards the tip of the needle, without requiring the patient to hold a particular gaze [18]. Anecdotally, some ophthalmologists at our institution recommend constant digital “fibrillation” of the globe during injection to disrupt any potential tethering of the needle to the optic nerve sheath. Intrasheath injection is also associated with a 3-4-fold increase in back pressure; therefore, any uneven or excess resistance should prompt needle retraction and redirection [3, 16, 22, 23].

Although no large, controlled comparisons have been made between needle tips, some authors have suggested the use of a blunt needle in order to reduce the chance of optic nerve penetration. Even so, several cases of BSA have still occurred with the use of blunt needles. Nicolls et al., after switching to blunt needle tips, felt that the risk of BSA with sharp tips did not outweigh the demonstrably greater pain and tissue damage associated with blunt injection [15]. Blunt needles also appear to result in worse visual acuity outcomes in cases in which accidental globe perforation does occur [17]. The risk of globe perforation, however, is even rarer (0 out of 44,000 injections), than BSA itself, even with shorter <31 mm needle tips, which have widely replaced the historical 38 mm length [24].

While some institutions and practitioners continue to favor single-agent injections, as was the case here, central nervous system (CNS) depression is a well-known complication of bupivacaine monotherapy. Rodman et al. reported a BSA rate of 1.5% over 200 injections when using bupivacaine 0.75% alone (Table 1), and a complete cessation of CNS complications after bupivacaine was mixed with other anesthetic components [19]. Lidocaine 4% is also associated with a ninefold increased risk for respiratory depression when compared to lidocaine 2%. The same study, which involved measuring serum levels of anesthetic after retrobulbar injection, found that larger volumes of injected anesthetic was also associated with increased risk for systemic spread. The authors advised using lidocaine 2% and the minimal amount of volume needed to achieve the intended effect [8]. Similarly, hyaluronidase is thought to increase the chance for BSA by enhancing tissue spread, and some authors have recommended a max dose of just 15 IU [25].

Aside from supportive/preventive care, when cases of BSA do develop, the American Society of Regional Anesthesia and Pain Medicine has included the use of lipid emulsion therapy (LET) among their published safety guidelines. Lipid emulsion functions as a “lipid sink,” by remaining separate from the plasma and encapsulating any circulating lipophilic toxin. The recommended treatment regimen is an initial bolus of 20% lipid emulsification at a dose of 1 mg/kg over 1 minute, followed by 15 mL/kg/hr. LET is most commonly used to reverse cardiotoxicity, usually in the context of dental, lumbar, or lower extremity nerve blocks [26]. Support for LET in the ophthalmologic literature is sparse, especially for cases with neurologic manifestations. Several cases, however, have shown a reversal of neurologic symptoms with the use of LET, including in one patient who received a retrobulbar block [27, 28]. Because of its rarity in the ophthalmologic setting, only 45% of practicing ophthalmologists are even aware of LET as a treatment option for systemic toxicity [29].

## 4. Conclusion

While few recommendations have reached a generalized consensus in the literature, several of the methods outlined above appear to be reasonable suggestions for both the early identification and prevention of BSA. Observation of the contralateral eye, performance of the block prior to surgical draping, a central globe position, shorter needle tips, judicious volume use, and mixed formulations are all viable practice-changing options for reducing the risk of BSA in patients undergoing eye surgery. Although over 70% of ophthalmologists use localized anesthetics on a regular basis, few are aware of lipid emulsion therapy as a potential reversal agent. The quick availability of LET in the outpatient or ambulatory care setting is even less certain. Further research on the utility of LET in the ophthalmologic setting is needed in order to determine if LET should be a part of standard education in ocular anesthesia.

## Figures and Tables

**Table 1:** Prevalence of brainstem anesthesia after retrobulbar block.

Study	# of injections	Prevalence (%)
Nicoll et al. (1987) [17]	6000	0.27
Wittpenn et al. (1986) [8]	3123	0.79
Ahn et al. (1987) [16]	∼3000	0.27
Rodman (1987) [19]	200	1.5
